# Prophylactic Anti-Osteoporotic Effect of *Matricaria chamomilla* L. Flower Using Steroid-Induced Osteoporosis in Rat Model and Molecular Modelling Approaches

**DOI:** 10.3390/antiox11071316

**Published:** 2022-07-01

**Authors:** Abirami Raja, Govind Pratap Singh, Sana A. Fadil, Sameh S. Elhady, Fadia S. Youssef, Mohamed L. Ashour

**Affiliations:** 1 Department of Pharmacology, Himalayan Pharmacy Institute, Majitar, Rangpo 737136, Sikkim, India; 2 Department of Chemistry, School of Basic Sciences, Shri Ramasamy Memorial University Sikkim, Gangtok 737102, Sikkim, India; govindpratapsingh.v@srmus.edu.in; 3 Department of Natural Products, Faculty of Pharmacy, King Abdulaziz University, P.O. Box 80260, Jeddah 21589, Saudi Arabia; safadil@kau.edu.sa (S.A.F.); ssahmed@kau.edu.sa (S.S.E.); 4 Department of Pharmacognosy, Faculty of Pharmacy, Ain-Shams University, Abbasia, Cairo 11566, Egypt; fadiayoussef@pharma.asu.edu.eg; 5 Department of Pharmaceutical Sciences, Pharmacy Program, Batterjee Medical College, P.O. Box 6231, Jeddah 21442, Saudi Arabia

**Keywords:** Asteraceae, corticosteroid, ADME/TOPAKT, *Matricaria* *chamomile*, molecular docking, osteoporosis, drug discovery, health care

## Abstract

The anti-osteoporotic activity of ethanol extract from the *Matricaria chamomilla* L. flower was evaluated using steroid-induced osteoporosis in a rat model for the first time. Biochemical parameters such as serum calcium, phosphate, magnesium, creatinine, and alkaline phosphatase were assessed. At a 400 mg/kg body weight dose, the extract showed 54.01% and 27.73% reduction in serum calcium and phosphate ions serum levels, respectively. Meanwhile, it showed a 20% elevation in serum magnesium level, compared to the steroid-treated group. It also showed a significant decrease in creatinine and alkaline phosphatase levels, by 29.41% and 27.83%, respectively. The obtained results were further supported by biomechanical analyses, which revealed that a 400 mg/kg body weight dose of the flower extract increased bone strength and thickness. At the same time, it does not affect the bone length, compared to the diseased group. Histopathological examination revealed that the extract showed a significant increase in trabecular thickness, and it had restored the architecture of the cortical and trabecular structure with well-organized bone matrix. The possible inhibitory effect of the major phenolic compounds identified from the plant extract on cathepsin K was investigated using molecular docking. Rutin (4) had the best-fitting score within the active site, as evidenced by the free binding energy, (∆G = −54.19 Kcal/mol). ADMET/TOPKAT revealed that the examined compounds had variable pharmacodynamics and pharmacokinetic properties that could be improved to enhance the bioavailability during incorporation in various dosage forms. Thus, it can be concluded that this plant extract showed potential therapeutic benefits for osteoporosis.

## 1. Introduction

Osteoporosis is defined as a “silent disorder” characterized by decreased bone mineral density associated with altered microarchitecture that leads to fracture with less or no trauma [1]. Osteoporosis includes vertebral compression, Colles’ distal radial fracture, kyphosis, and hip fracture [2]. The qualitative abnormalities in bone quality are characterized by altered matrix composition and bone mass, altered cement lines, and cortical porosity with trabecular disruption. The major fracture sites are vertebral bodies, hipbone, distal radius, and proximal femur [3].

This skeletal disorder represents a major public health problem threatening all ethnic groups. The incidence of osteoporosis was found to be more common in women than men [4]. Various causes trigger osteoporosis, including genetics, unhealthy lifestyle, aging, postmenopausal effect, lack of physical exercise, and dietary impairment [5]. Anyway, diseases such as hyperthyroidism, anorexia, hyperlipidemia, surgical removal of ovaries, kidney disease, excess alcoholism, and reduced levels of estrogen and androgen hormones are considered among the major reasons for developing of osteoporosis. Prolonged usage of some medications such as anti-epileptics, anti-neoplastic, proton pump inhibitors, selective serotonin reuptake inhibitors, and steroids, can cause osteoporotic conditions [6,7].

Many antiresorptive and anabolic therapies are commonly used to ameliorate osteoporosis [8]. Antiresorptive therapies that decreases the rate of bone loss includes bisphosphonates, estrogen replacement therapy, and selective estrogen receptor modulators. These drugs prevent osteoclast-mediated bone loss and reduce bone turnover [9]. Meanwhile, drugs that promote bone formation include calcium, vitamin D analogs, and teriparatide [10]. Although most of these drugs successfully ameliorate osteoporosis, they can produce undesirable side effects. Therefore, the search for safer, naturally-occurring, biologically-active entities is vital worldwide [11].

*Matricaria chamomilla* (Asteraceae) is native to Europe and western Asia and widespread worldwide. The name chamomile is derived from the Greek language meaning “Ground Apple” because of its odor. Meanwhile, Ancient Egyptians considered this plant a gift of the Almighty [12]. In Europe, the plant is used for many diseases “one plant cures all”, with a long history in Germany [13]. Around the fifth century, the plant was used for spinal pain, nerve pain, rheumatoid arthritis, indigestion, sunstroke, fever, flatulence, vasodilation, and insomnia. In addition, it is consumed as a tea to treat stress and sleeplessness, and its oil was used to treat cystitis, flu, infections, malaria, and eczema [14]. This considerable biological activity may be attributed to its richness in secondary metabolites, particularly terpenoids, flavonoids, coumarins, Spiro ethers, and tannins [15].

Herein, we aimed to investigate the anti-osteoporotic activity of the ethanol extract of the *Matricaria chamomilla* L. flower using steroid-induced osteoporosis in the rat model for the first time. This study was planned based on the richness of the *Matricaria chamomilla* L. flower with many flavonoids, particularly apigenin, which plays a major role in the differentiation and growth of osteoblast cells, as well as bone formation and resorption processes in oxidative stress-induced osteoporosis [6]. Anyway, biochemical parameters such as serum calcium, phosphate, magnesium, creatinine, and various biomechanical analyses, such as bone breaking strength, thickness, and length, were assessed. These results were further supported by the histopathological examination of the femur bone. Molecular docking was performed by Discovery Studio^®^ 4.5 (Accelrys Inc., San Diego, CA, USA) on the previously identified secondary metabolites from the plant on the cathepsin K enzyme. This protease has been recognized as a novel therapeutic target to treat osteoporosis and other disorders characterized by an elevation in bone resorption. Previous studies showed that flavonoids effectively inhibited cathepsin K, particularly luteolin, which significantly inhibited osteoclast-associated genes comprising cathepsin K [16,17]. Additionally, the evaluation of different pharmacodynamic, pharmacokinetic, and toxicity parameters was performed via ADME/TOPKAT postulation.

## 2. Materials and Methods

### 2.1. Plant Material

*Matricaria chamomilla* Linn (Asteraceae) is widely distributed in the Himalayan ranges, and the flowers were procured from Agastia Pharma, Mumbai. After procuring the plant, the flowers were authenticated, shade-dried, and powdered for extraction. The plants were kindly identified and authenticated by Professor Sasikala Ethirajulu, Consultant of Pharmacognosy at the Central Council for Research in Siddha Chennai, Ministry of AYUSH, and the Government of India. Voucher specimens of the authenticated plant, with codes AR-P-Mc-1, were kept at the Department of Pharmacology, Himalayan Pharmacy Institute, Majitar-737136, India.

### 2.2. Preparation of the Plant Extract

A total of 150 g of the dried *Matricaria chamomilla* flowers were used to prepare the extract by cold maceration with 90% *v/v* ethanol (3 × 500 mL) and occasional shaking at room temperature for seven days [18]. Then, the extracted contents were decanted on a sterilized muslin cloth. The extract was then dried using a vacuum rotary evaporator at 45 °C and lyophilized to give 42 g of dried extract, which was stored in a desiccator for further study at 25 °C.

### 2.3. In Vivo Anti-Osteoporotic Activity Evaluation

#### 2.3.1. Animals and Animal Treatment

Wistar rats of either sex weighing 200–220 g of 5–6 months old were chosen for the study. The animals were housed in polypropylene cages, and paddy husk was used as bedding. Animals were maintained at the temperature of 25 ± 2 °C, following a 12 h light/dark cycle. Animals were provided with a standard pellet diet and water ad libitum. The weight of the animals was noted prior to the study. The experimental protocol received approval from of Institutional Animal Ethical Committee, number IAEC/MMC/17/2016.

#### 2.3.2. Experimental Protocol

A glucocorticoid-induced osteoporotic rat model was used, where methyl prednisolone (representative corticosteroids) was chosen to be administered for inducing osteoporosis in rats at a dose of 28 mg/kg, through subcutaneous route, once a week for four weeks. The activity and potency of the tested samples were compared to alendronate, a standard bisphosphate drug used to treat osteoporosis, especially when the osteoporosis is induced in the premenopausal phase or on prolonged use of steroids. Alendronate was administered orally at a dose of 2 mg/kg daily for eight weeks [19]. Anyway, the doses of the plant extract for this study were chosen as 1/5th and 1/10th of the toxic dose, previously determined to be 2000 mg/kg, as reported by Karbalay-doust et al. [18]. Hence, the animals in the test groups were administered doses of 200 and 400 mg/kg b.wt, where the dried lyophilized ethanol extract was suspended in carboxy methyl cellulose and administered orally as an oral suspension for eight weeks. The animals were classified randomly into five groups, each containing 6 rats. In the first group (normal control), the animals received normal diet and water ad libitum; meanwhile, in the second group (diseased group), the rats were injected subcutaneously with 28 mg/kg b.wt of methylprednisolone once a week from the 4th–8th week. In the third group (standard group), alendronate was administered orally in a dose of 2 mg/kg b.wt for eight weeks, in addition to subcutaneous administration of 28 mg/kg b.wt of methylprednisolone once a week from the 4th–8th week. In the fourth and fifth groups, the tested extracts were administered in doses of 200 and 400 mg/kg b.wt, respectively, for eight weeks, together with subcutaneous administration of 28 mg/kg b.wt of methylprednisolone once a week from the 4th–8th week. In cases of oral administration, oral gavage using oral feeding tube fitted with a syringe needle was used. At the end of the eighth week, the weight of each animal was measured, samples were aseptically collected, and anti-osteoporotic effect on the animal was evaluated by performing biochemical, biomechanical, and histopathological assessments. The blood was collected from retro-orbital plexus after anaesthetizing the animals’ intraperitoneal administration of 23 mg/kg b.wt thiopentone sodium using a fine capillary tube. The collected blood was then processed by centrifugation at 3000 rpm for 15 min. to separate serum from cells and stored at a temperature of −20 °C.

#### 2.3.3. Evaluation of the Biochemical Parameters

The serum level of calcium, inorganic phosphorus, and magnesium were evaluated spectrophotometrically. The serum inorganic calcium was quantitatively evaluated using colorimetric end-point method, in which the calcium is reacted with *o*-cresolpthalein and produced a purple complex at pH 10–12 with an absorbance maximum of λ = 575 nm. The intensity of the measured color is directly proportional to the calcium concentration. In addition, inorganic phosphate reacted with ammonium molybate at acidic pH to produce a blue color complex of ammonium phosphomolybdate at λ = 570 nm. However, when magnesium reacted with a metallochromic dye called calmagite, it forms calmagite magnesium complex, which gives a pink color at λ = 520 nm. Meanwhile, serum alkaline phosphatase catalyzes the hydrolysis of *p*-nitrophenyl phosphate (pNPP) to *p*-nitrophenol, which is colorless; however, it has a strong absorbance at λ = 405 nm; thus, the rate of the increased absorbance is proportional to enzyme activity [20]. The kits used to evaluate of various biochemical parameters are as follows.

Serum inorganic calcium was quantitatively evaluated using colorimetric end-point method via calcium colorimetric assay kit (MAK 022-1KT; Sigma Aldrich, Steinheim, Germany). Serum inorganic phosphate was quantitatively evaluated using phosphate kit molybdate UV method (Cliniquant-FSR, Meril diagnostics, New Delhi, India). Serum inorganic magnesium was quantitatively evaluated using magnesium colorimetric assay kit (E-BC-K162-5; Elabscience, Hubei, China). Creatinine concentration is determined by a coupled enzyme reaction, which results in a colorimetric (570 nm) product proportional to the creatinine present using creatinine assay kit (MAK080, Sigma Aldrich, Steinheim, Germany). Serum alkaline phosphatase activity was determined by the principle, where *p*-nitrophenyl phosphate, which hydrolyzed by ALP into a yellow-colored product (maximal absorbance at 405 nm). The rate of the reaction is directly proportional to the enzyme activity, using alkaline phosphatase assay kit (MAK447; Sigma Aldrich, Steinheim, Germany).

#### 2.3.4. Evaluation of the Biomechanical Parameters

The processed femur bone was used to analyze the biomechanical strength of the bone. After euthanizing the animal by an overdose of thiopental sodium injection, the animal was placed on a dissecting board, and the hip ball and socket joint was reached by invasion and retraction. The bone was dislocated and cleansed by removing the tissues around it and stored in formalin. The femur was then assessed for the bone breaking strength, as well as the bone length, weight, and thickness of femur.

##### Determination of Bone Hardness

The hardness of the bone was measured, in order to determine the mechanical properties of the bone that reflects the bioactivity of the tested extract. The hardness was measured by determining the fracture point. The fracture point measures the point at which the bone breaks when weight is applied. In the present study, the Monsanto hardness tester was used. This bone-breaking strength is indicated as kg/cm^2^ unit [21].

##### Determination of Length, Weight, and Thickness of Femur

The length was measured using a ruler, whereas the thickness was measured at the epiphyseal growth plate region using a vernier caliper. The weight of each bone was measured using digital balance [21].

##### Histopathological Examination

The isolated femur bone was defatted by treating the bone with 5% nitric acid for 24 h. Then, the bone samples were dehydrated using an automated vacuum tissue processor. The dehydrated samples were embedded in paraffin histology wax and then sectioned. The sectioned bone was stained with hematoxylin and eosin (H&E) stains and observed under a light microscope [21].

#### 2.3.5. Statistical Analysis

All the values are expressed as mean ± SD. The data were statistically analyzed by one-way ANOVA, followed by Dunnett’s test. One-way ANOVA was used to correlate the statistical difference between the variables. *p* < 0.05 is considered significant.

### 2.4. In Silico Molecular Modeling Studies

#### 2.4.1. Molecular Docking

Molecular docking was performed on the major previously identified phenolic secondary metabolites from *Matricaria chamomilla* L., in order to examine their potential inhibitory effect on cathepsin K obtained from the protein data bank (PDB ID 1VSN; 2.00 Å), (http://www.pdb.org, accessed on 10 April 2022) [22]. This protease enzyme has been recognized as a novel therapeutic target to treat osteoporosis and other disorders that are characterized by an elevation in bone resorption. Assessment was carried out using C docker algorithm by Discovery Studio software 4.5 (Accelrys Inc., San Diego, CA, USA), following what was previously reported [23,24].

#### 2.4.2. ADME/TOPKAT In Silico Evaluation

The evaluation of different pharmacodynamic, pharmacokinetic, and toxicity parameters, such as absorption, distribution, metabolism, excretion, and toxicity, via ADME/TOPKAT postulation was performed using in silico approaches by Discovery Studio software 4.5 (Accelrys Inc., San Diego, CA, USA). Rat oral LD50, Ames mutagenicity, carcinogenic effect on male and female rat FDA, ocular and skin irritant effect, as well as rat chronic LOAEL (lowest observed adverse effect level) were selected as toxicity descriptors. For ADMET determination, penetration of blood–brain barrier (BBB), aqueous solubility, intestinal absorption in human, and binding prediction (PPB), as well as cytochrome P450 2D6 inhibition and hepatotoxicity level, were selected [25].

## 3. Results

### 3.1. In Vivo Anti-Osteoporotic Activity Evaluation

#### 3.1.1. Determination of the Biochemical Parameters

##### Effect of *Matricaria chamomilla* on Serum Calcium Level in Steroid Induced Osteoporotic Rat Model

**Figure 1:**
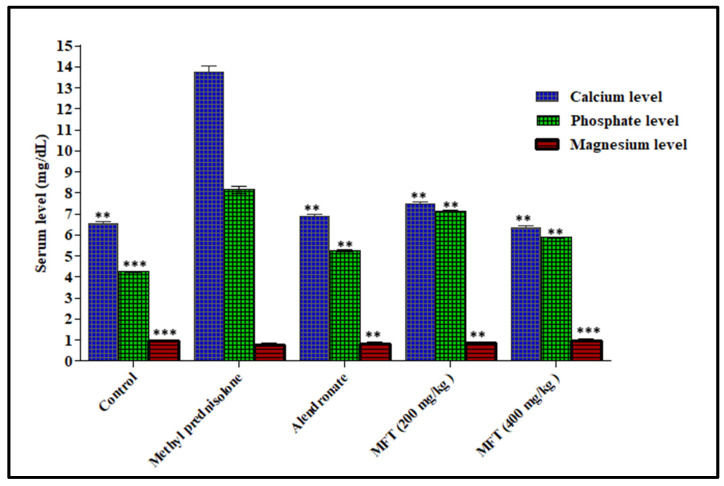
Effect of *Matricaria chamomile* Linn on serum calcium, phosphate, and magnesium levels of steroid-induced osteoporotic rat model. Values are expressed as mean ± SEM (*n* = 6), where ** *p* < 0.01, *** *p*< 0.001, as compared to diseased control.

Generally, the serum level of calcium represents a reflection of bone resorption activity. Thus, an increased serum calcium level is one of the parameters that confirm the osteoporotic condition. Administration of the steroid drug methylprednisolone drastically increased the serum calcium level in treated animals by 110.77%, compared to the normal control group. Meanwhile, the oral administration of the tested extracts significantly ameliorated the osteoporotic condition, as evidenced by lowering of serum level of calcium by 45.26% and 54.01%, compared to the steroid drug-treated animals upon treatment with 200 and 400 mg/kg b.wt, respectively. These results are closely similar to those of alendronate, the standard drug, which showed a 50.36% reduction in serum calcium, compared to the steroid-treated group (Figure 1). Hence, it is obvious that the tested samples revealed a significant effect on bone resorption activity, as seen by the serum calcium level, where remarkable healing was observed, particularly at 400 mg/kg b.wt.

##### Effect of *Matricaria chamomilla* on Serum Phosphate Level in Steroid Induced Osteoporotic Rat Model

Phosphate is one of the most important minerals crystallized with calcium as hydroxyapatite salt in bones. During resorption mediated by parathyroid hormone, the serum phosphate level increases, which denotes the progression of the osteoporotic condition. The serum phosphate level was significantly elevated upon treating the animals with methylprednisolone by 94.04%, relative to the normal control group, illustrating active bone resorption and poor bone formation. In contrast, oral administration of 200 and 400 mg/kg b.wt of the ethanol extract of *Matricaria chamomilla* flowers showed a promising anti-osteoporotic activity, as manifested by the pronounced decline in the serum phosphate level by 12.63% and 27.73%, respectively, relative to the steroid-treated animals. Meanwhile, oral administration of alendronate, the standard drug, showed a 35.58% reduction in serum phosphate, compared to steroid-treated rats. Thus, the tested extracts exhibited a promising anti-osteoporotic activity, enhanced phosphate deposition, and healthy bone formation, but it was at a slightly lower level than alendronate (Figure 1).

##### Effect of *Matricaria chamomilla* on Serum Magnesium Level in Steroid Induced Osteoporotic Rat Model

The trace element magnesium is responsible for the hydroxyapatite crystal formation and conversion of vitamin D to its active form. The decreased serum magnesium level indicates the development of the osteoporotic condition, as magnesium is important for bone formation. Results illustrated in Figure 1 showed that the administration of methylprednisolone reduced the serum magnesium level by 20.83%, whereas the alendronate-treated group elevated the serum magnesium level by 5.26%, compared to the normal control group. The diseased control group showed a lesser magnesium level because of free radical formation and impaired absorption of magnesium from the dietary source, which was mediated by the steroid. Meanwhile, notable amelioration was observed upon treating the animals with 200 and 400 mg/kg b.wt of the ethanol extract of *Matricaria chamomilla* flowers, which resulted in 14.47% and 20.00% elevation in serum magnesium and restored it back to the normal level.

##### Effect of *Matricaria chamomilla* on Serum Creatinine Level in Steroid Induced Osteoporotic Rat Model

**Figure 2:**
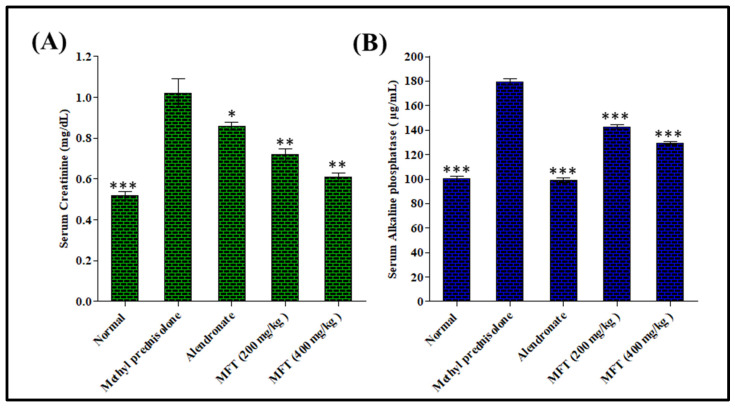
Effect of *Matricaria chamomilla* flowers total ethanol extract (MFT) on serum creatinine (mg/dL) (**A**) and alkaline phosphatase level (µg/mL) (**B**) on steroid-induced osteoporotic rat model. Values are expressed as mean ± SEM (*n* = 6). Significantly different from the diseased group at *** *p* < 0.001, ** *p* < 0.01, and * *p* < 0.05.

Creatinine acts as a hydroxyl radical scavenger; therefore, the higher the creatinine level, the greater the chance of bone resorption. From the above results in Figure 2, it was obvious that the disease control group treated with the steroid showed an increased serum creatinine level by 96.15%, compared with the normal control group. The serum creatinine level in the standard treated group decreased by 40.20%, compared to the disease-treated group. However, the test drug-treated groups at doses of 200 and 400 mg/kg b.wt showed a significant decrease in creatinine level by 15.69% and 29.41%, compared to the diseased control group.

##### Effect of *Matricaria chamomilla* on Serum Alkaline Phosphatase Level in Steroid-Induced Osteoporotic Rat Model

The serum alkaline phosphatase level acts as an indicator of excessive bone activity, where an increased level represents an increased bone activity, leading to increased bone loss in the future. The results, illustrated in Figure 2, show that the diseased control group exhibited an increased level of serum alkaline phosphatase estimated at 79.02%, compared to the normal control group. In contrast, the alendronate-treated group significantly decreased serum alkaline phosphatase level by 44.74%, compared to diseased group. Furthermore, the sample-treated groups in doses of 200 and 400 mg/kg b.wt showed significant decreases in alkaline phosphatase by 20.41% and 27.83%, respectively, compared to the diseased-control group. The ethanol extract of *Matricaria chamomilla* flowers significantly decreased the alkaline phosphatase level in a dose-dependent manner, where treatment in a dose of 400 mg/kg b.wt showed values close to the standard and normal control groups.

#### 3.1.2. Determination of the Biomechanical Parameters

The bone mechanical properties were assessed via the determination of the cortical and trabecular strength and bone mineralization. The fracture point was determined by applying weight to the femur bone. It was found that the diseased group receiving methylprednisolone only experienced increased bone porosity and was broken with less weight, compared to the normal control group. On the contrary, the ethanol extract of *Matricaria chamomilla* flowers showed increased bone strength, compared to diseased animals, when administered at a dose of 400 mg/kg b.wt, which is close to the standard and normal groups. It is worthy of mentioning that, while there was no significant change in bone length across groups, the test drug group had greater thickness, compared to the disease control group (Table 1).

#### 3.1.3. Histopathological Examination

**Figure 3:**
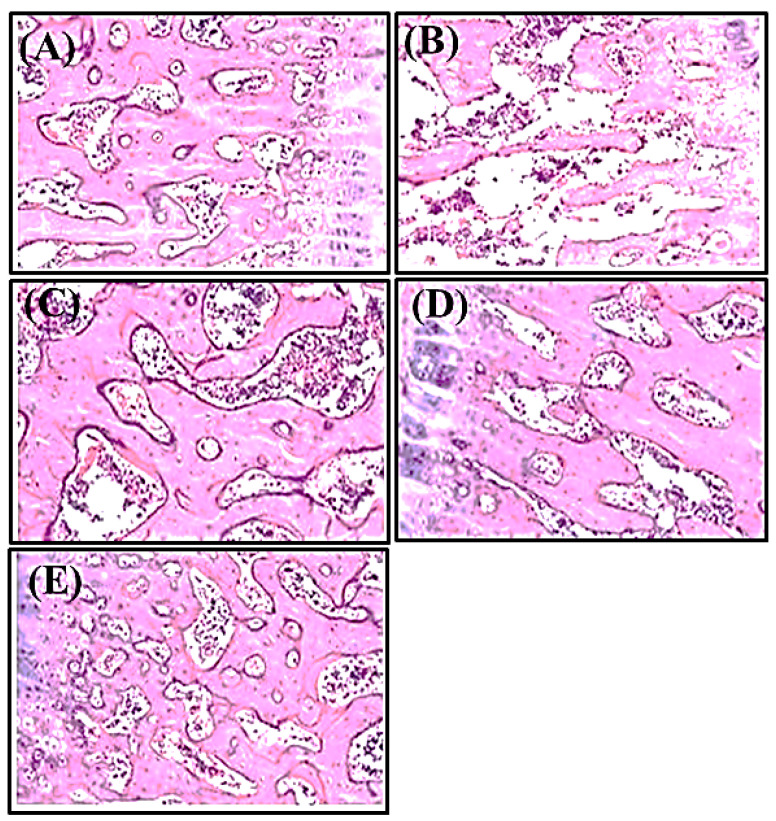
Histopathology of femur bone which was observed under (40×) magnification (**A**) Normal control, (**B**) diseased control only, treated with methyl prednisolone (28 mg/kg, s.c once a week for four weeks), (**C**) standard control, treated with alendronate (2 mg/kg, p.o for 8 weeks) and methyl prednisolone (28 mg/kg, s.c once a week for four weeks), (**D**) MFT (200 mg/kg p.o for 8 weeks) along with prednisolone (28 mg/kg, s.c once a week for four weeks), (**E**) MFT (400 mg/kg p.o for 8 weeks) along with prednisolone (28 mg/kg, s.c once a week for four weeks).

The results illustrated in Figure 3 demonstrate the effect of *Matricaria chamomile* L. on bone histology and structure, as well as the trabecular thickness, osteoid, and eroded region over the surface of the femur bone. Trabecular thinning was noticed in the diseased group administered with the steroid only, which may be attributed to the elevation in osteoclastic cells number, decrease in osteoblastic cells, and increased resorption of bone in disease control group. Comparing the steroid-treated group with the alendronate-treated control group revealed a significant improvement, regarding the increase in trabecular thickness and normalization of architecture of the cortical and trabecular structure with the well-organized bone matrix. The standard control group showed improved trabecular thickness, bone architecture, and histology. However, comparing the *Matricaria chamomilla*. L.-treated groups with the diseased group, the formers showed significant increase in trabecular thickness, with a well-organized bone matrix, which may be due to an increase in the osteoblastic cell number with a concomitant decrease in the osteoclastic cell number. It is worth highlighting that the *Matricaria chamomilla* extract-treated group at a dose of 400 mg/kg b.wt showed close activity to alendronate and reduced trabecular space. Therefore, it is obvious that the ethanol extract of *Chamomile* flowers is active in bone formation and has pronounced therapeutic benefits to treat osteoporosis.

### 3.2. Functional Phenolic Substances Predominating in Matricaria chamomilla Flowers

**Figure 4:**
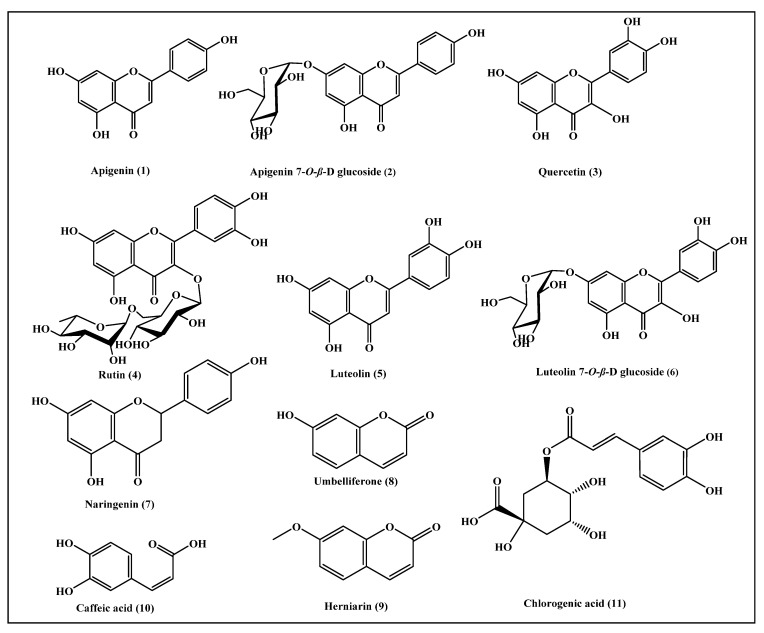
Scheme showing the chemical structures of phenolic bioactive compounds previously identified from *Matricaria chamomilla* flowers.

The flower of *Matricaria* *chamomilla* represents a rich source of chemical compounds that possess potential therapeutic activities. About 120 secondary metabolites were identified from *Matricaria chamomilla* flowers, mostly belonging to coumarins, flavonoids, sesquiterpenes, and polyacetylenes. Furthermore, the flower is a rich source of essential oil, which is rich in sesquiterpene derivatives, together with traces of monoterpenes and polyynes, such as α-bisabolol and its oxide, in addition to chamazulene, farnesene, farnesol, matricin, isobutyl angelate, and 2-methylbutyl angelate. Anyway, eleven phenolic bioactive compounds were previously identified from *Matricaria chamomilla* flowers, namely apigenin (1), apigenin-7-*O*-β-glucoside (2), quercetin (3), rutin (4), luteolin (5), luteolin-7-*O*-β-glucoside (6), naringenin (7), umbelliferone (8), herniarin (9), caffeic acid (10), and chlorogenic acid (11) [26,27,28,29,30,31], which are illustrated in Figure 4.

### 3.3. In Silico Molecular Modeling Studies

#### 3.3.1. Molecular Docking

The plant was documented to have a high concentration of flavonoids, particularly apigenin, which play a major role in the differentiation and growth of osteoblast cells, as well as in the bone formation and resorption process in oxidative stress-induced osteoporosis [6]. Thus, molecular docking was performed on previously identified major phenolic secondary metabolites obtained from *Matricaria chamomilla* L., in order to examine their potential inhibitory effect on cathepsin K. This enzyme has recently been recognized as a therapeutic target to treat osteoporosis and other disorders that are characterized by an elevation in bone resorption [22]. The results illustrated in Table 2 show that all of the examined phenolic compounds showed considerable inhibitory activity on cathepsin K to varying degrees.

**Figure 5:**
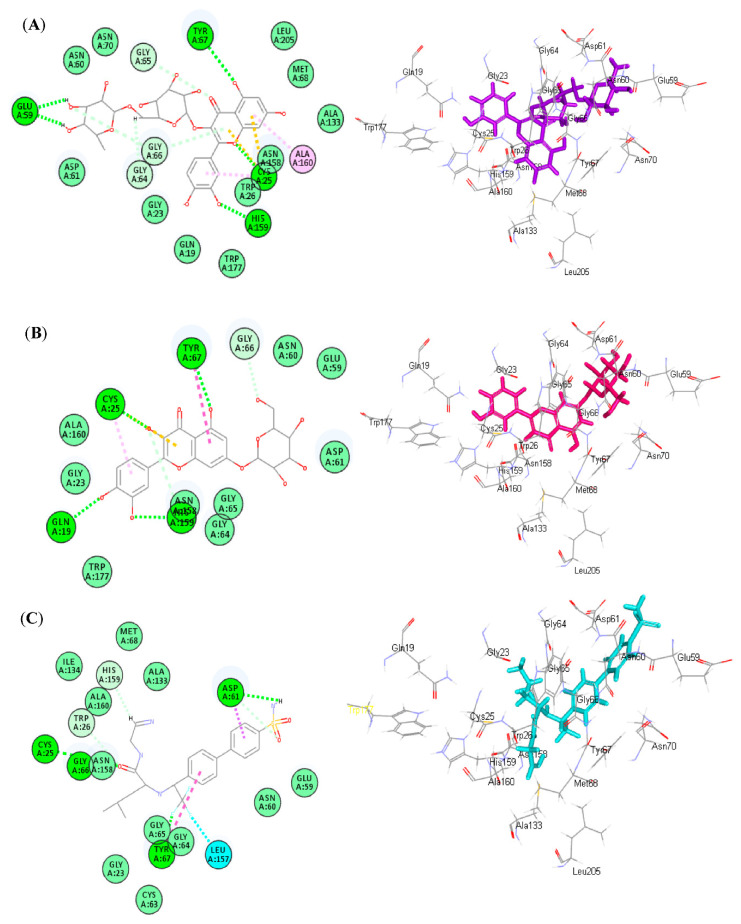
The 2D and 3D binding modes of rutin (4) (**A**), luteolin-7-*O*-β-glucoside (6) (**B**), and Co-crystalized ligand (NFT) (**C**) inside the active center of the active sites of cathepsin K; heavy green dotted bond, H-bonds; heavy pink dotted bond, π-π bonds; light green dotted bond, C-H bonds; light pink dotted bond, π-alkyl bonds; purple dotted bond, π-δ bond; orange dotted bonds, π-sulphur bond; blue dotted bond, halogen bond.

Rutin (4) showed the best fitting activity within the active site of the tested protein, followed by luteolin-7-*O*-β-glucoside (6) and apigenin-7-*O*-β-glucoside (2), as evidenced by their free binding energies (∆G) with values −54.19, −41.91, and −39.57 Kcal/mol, respectively, approaching that of the co-crystallized ligand (∆G = −67.16 Kcal/mol). Rutin (4) forms several bonds with the amino acid residues existing at the binding sites comprising five conventional H-bonds with Glu59, Tyr67, Cys25, His159, and the hydroxyl moieties in the active entity. Furthermore, it forms two tight π-sulphur bonds between the aromatic moieties of the compound and Cys25 amino acid, as well as four C-H bonds with Gly64, Gly65, and Gly66, together with one π-alkyl bond with Ala160 (Figure 5A). Regarding luteolin-7-*O*-β-glucoside (6), it forms four H-bonds with Cys25, Tyr67, His159, Gln19, as well as one tight π-sulphur bond with Cys25 amino acid, one π-π bond with Tyr67, one π-alkyl bond with Cys25, in addition to two C-H bonds with Gly66 and Asn158 (Figure 5B). However, apigenin-7-*O*-β-glucoside (2) forms two H-bonds with Gln19 and Trp177, as well as one tight π-sulphur bond with His159, one C-H bond with Gly64, in addition to two π-donor H-bond interactions with His159 and Cys25. Meanwhile, the co-crystallized ligand with the protein forms three H-bonds with Cys25, Gly66, Asp61, and Tyr67, as well as three C-H bonds with His159, Trp26, and Asp61, one halogen bond between fluorine moiety in the compound and Leu156, and one π-π bond with Tyr67, in addition to one π-δ bond with Asp61 (Figure 5C).

#### 3.3.2. ADME/TOPKAT In Silico Evaluation

Different pharmacodynamic and pharmacokinetic properties, such as absorption, distribution, metabolism, and excretion and toxicity parameters, were determined for the major phenolic compounds previously identified from the *Matricaria chamomilla* L. flower, which are illustrated in Figure 4, using in silico approaches. Regarding human intestinal absorption, most of the examined compounds revealed good to moderate absorption levels, taking the values of 0 and 1, respectively, except for the flavonoid glycosides.

**Figure 6:**
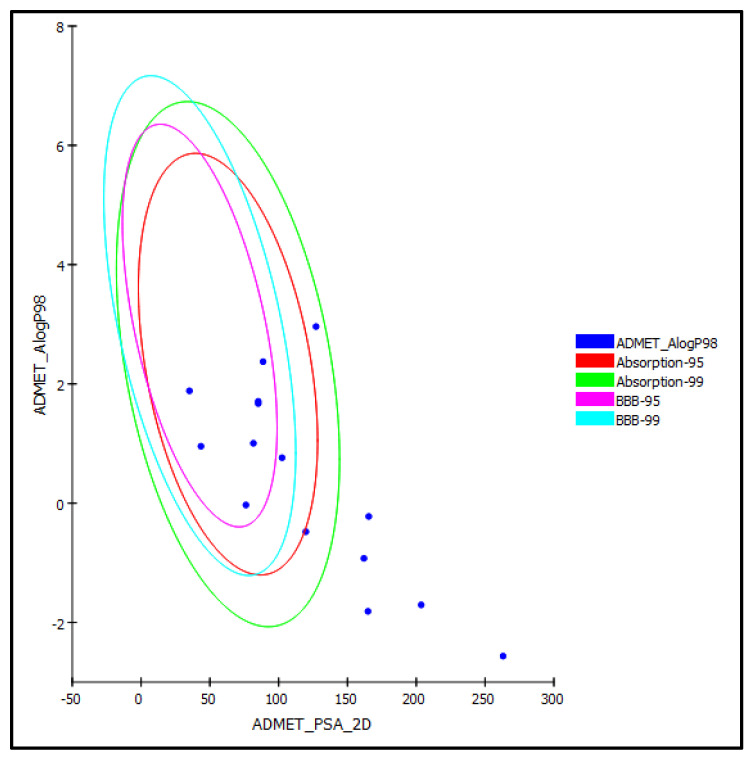
ADMET plot for of phenolic bioactive compounds previously identified from *Matricaria chamomilla* flowers, showing 95% and 99% confidence limit ellipses, corresponding to the blood-brain barrier (BBB) and human intestinal absorption models in ADMET_AlogP98.

Apigenin-7-*O*-β-glucoside (2), rutin (4), luteolin-7-*O*-β-glucoside (6), and chlorogenic acid (11) revealed very low absorption levels, due to their highly polar nature; thus, they were located outside the 99% absorption ellipses, as displayed in the ADMET plot (Figure 6).

Similarly, together with co-crystalized ligand (NFT) and quercetin, they showed undefined penetration via BBB with level 4 and, hence, were placed outside the 99% BBB confidence ellipse, whereas the rest of compounds showed medium to low BBB penetration and, thus, were located within the 99% of BBB confidence ellipse. Regarding the solubility level, all of the tested compounds showed good to optimal solubility, in contrast to the co-crystalized ligand (NFT), which revealed very low, but possible, solubility. Concerning the plasma protein binding pattern (PPB) that is crucially important in the determination of pharmaceutical activity, apigenin (1), quercetin (3), luteolin (5), and herniarin (9), as well as the co-crystalized ligand (NFT), showed more than 90% plasma protein binding, whereas the rest of compounds showed a PPB of less than 90%. Additionally, the results illustrated in Table 3 showed that all of the tested compounds, except herniarin (9), caused no inhibition to cytochrome P450 2D6 (CYP2D6), which is incorporated in the metabolism of many xenobiotics, thus causing unpredictable drug–drug interactions. However, all of the compounds showed certain hepatotoxicity, in contrast to the phenolic acids, namely caffeic acid (10) and chlorogenic acid (11) are non-hepatotoxic (Table 3).

For TOPKAT prediction displayed in Table 4, it is obvious that all of the tested compounds revealed no mutagenicity in Ames mutagenicity in silico study. Anyway, they revealed mild to no skin irritant effect, whereas they caused mild to moderate eye irritation. Furthermore, all of the compounds showed non-carcinogenic effect to rat male FDA, except umbelliferone (8), herniarin (9), and chlorogenic acid (11), which showed certain carcinogenicity. Similarly, apigenin (1), quercetin (3), luteolin (5), and naringenin (7) showed certain carcinogenicity in rat female FDA and, thus, should be used cautiously. However, the tested phenolic compounds showed rat oral LD_50_ in the range of 0.206 to 1.363 g/kg b.wt, where quercetin exhibited the lowest rat oral LD_50_, in contrast to chlorogenic acid, which showed the highest value. Additionally, the tested compounds showed LOAEL values for the compounds ranging between 0.012 to 0.142g/kg b.wt, where the lowest value was displayed by apigenin-7-*O*-β-glucoside; meanwhile, the highest value was detected by quercetin.

## 4. Discussion

The anti-osteoporotic activity of the ethanol extract of *Matricaria chamomilla* flowers in a steroid-induced osteoporotic rat model was investigated for the first time. This study was planned based on the richness of *Matricaria chamomilla* L. flower with many flavonoids, particularly apigenin, which plays a major role in the differentiation and growth of osteoblast cells, as well as in the bone formation and resorption process in oxidative stress-induced osteoporosis. The main objective of the study was to find whether flavonoids and phenolic constituents of *Matricaria chamomilla* flowers prevents and protects bone from the oxidative stress caused by corticosteroids-mediated osteoporosis. The drug treatment given to the animals was planned prophylactically, and the study lasted eight weeks. Ethanol flower extract was given orally for the entire eight weeks at dose concentrations of 200 and 400 mg/kg, and methylprednisolone was given subcutaneously from the fifth to eighth weeks at a dose of 28 mg/kg. Although steroids exert various beneficial effects and are used as lifesaving drugs in several fatal conditions, when they are taken daily, they cause severe bone resorption, such as in patients who undergo organ transplantation or those who have severe asthma, arthritis, pneumonia. This induces osteoporosis as one of various side effects, which is non-symptomatic until single or multiple bone fractures occur [32].

Chamomile flower was considered a cure-all for diseases and was included in the pharmacopeia of many countries. The major constituents of chamomile flowers are alpha bisabolol, sesquiterpenes, coumarins (such as herniarin and umbelliferone), phenylpropanoids (such as chlorogenic acid and caffeic acid), flavones (such as apigenin, luteolin, quercetin, and rutin), and polyacetylene [26,27,28,29,30]. Biochemical and histopathological parameters were evaluated, in order to demonstrate the effect of plant extracts on osteoporosis. The obtained results confirmed the accumulative positive effects of flavonoids and other constituents of chamomile flower on bone microarchitecture and in the restoration of bone minerals.

Alkaline phosphatase is a hydrolase enzyme responsible for removing phosphate groups from many types of molecules, including nucleotides, proteins, and alkaloids; hence, the process of removing phosphate groups is called dephosphorylation, and it is considered an important bone turnover marker [33]. In humans, alkaline phosphatase is present in almost all the tissues throughout the entire biological system, but it is particularly concentrated in the liver, bile duct, kidney, bone, intestinal mucosa, and placenta. In the serum, two types of alkaline phosphatase predominate, namely skeletal alkaline phosphatase and hepatic alkaline phosphatase. In childhood stages, most of alkaline phosphatases are of skeletal origin [21]. Alkaline phosphatase is a chromosomal enzyme whose primary function is bone matrix mineralization [33]. The major biological process of this enzyme is osteoblastic cell differentiation, bio-mineral tissue development, cementum mineralization, cellular response to organic cyclic compound, skeletal system structural development, dephosphorylation, response to vitamin D, response to antibiotics, the developmental process in fetus, response to glucocorticoid, and endochondral ossification [34].The enzyme alkaline phosphatase level is high in serum when people have untreated coeliac disease, biliary obstruction, liver disease, leukemia, lymphoma, sarcoidosis, and myocardial infarction, as well as in bone diseases such as Paget’s disease, osteomalacia, osteoblastic bone tumors, and osteoporosis [35]. From the serum obtained from the rats in the study, *Matricaria chamomile* Linn flower extracts had a significant effect on alkaline phosphatase level. Serum calcium and phosphorous concentration convey critical information regarding the bone activity. The calcium and phosphate ions have an intrinsic attraction towards bones and form a crystalline deposition on bones as hydroxyapatite. The function of this crystal is to store the calcium and phosphate ions that are absorbed from the intestine and maintain the compressional strength of bones. Pyrophosphate is responsible for initiating hydroxyapatite precipitation in other tissues, along with the maintenance of low physiologic concentrations of calcium and phosphate ions, where osteoblasts inhibit pyrophosphate activity in the bone, thus allowing for precipitation to happen. The elevated levels of these ions in serum reflect the osteoporotic condition [36]. Meanwhile, the serum’s magnesium levels reflect bone mineralization and resorption activity. Magnesium is a trace element, where 60% of it was deposited on the hydroxyapatite crystal in bone. Magnesium is responsible for many biochemical processes within the bone, mainly the conversion of vitamin D to its active form, which, in turn, enhances calcium phosphate absorption in bone. Magnesium deficiency directly causes osteoporosis by affecting the crystal formation, as well as the secretion and activity of the parathyroid hormone, and promotes the low grade inflammation that, consequently, increases osteoclasts activity [37]. The results illustrated in Figure 1 show the elevation of both serum calcium and phosphate levels, with a concomitant decline in the serum magnesium level in animals receiving methyl prednisolone, which, in turn, reflects the oxidative stress-induced bone resorption triggered by the administered steroids. In contrast, *Matricaria chamomilla* Linn flower extracts showed a considerable reduction in serum calcium and phosphate levels, with concomitant elevation in the serum magnesium levels, compared to the steroids-treated group and approaching the normal control group.

Additionally, serum creatinine level represents another serum bone marker that inhibits bone activity. Increased serum creatinine indicates low bone mineral density (BMD) or increased bone resorption. The main pathogenesis for the effect of serum creatinine on BMD is that creatinine has a beneficial effect via acting as a hydroxyl radicle scavenger, which decreases oxidative stress and controls bone resorption. During oxidative stress-induced by steroid, there is an increase in proinflammatory mediators, such as cytokines and interleukins, which increase the osteoclastic differentiation, followed by the production of a great amount of free radicals, which make osteoclast cells matures to resorb the bone [38]. Furthermore, an increased free radical species affects and degrades the creatinine level, producing a positive feedback mechanism to enhance the osteoclast activity and increase resorption. Thus, the richness of *Matricaria chamomilla* flowers with phenolic compounds plays a crucial role in scavenging free radicals, thus promoting a reduction in bone resorption and restoring mineralization.

Moreover, the osteoporotic preventive effect of plant extract was also evidenced in the biomechanical parameters, such as bone breaking strength, bone thickness, and bone length. The results illustrated in Table 1 reflected the comparative differences in the bone breaking strength, length, and thickness. The bone breaking strength of the diseased control is less than the normal and standard control groups, where it showed the trabecular porosity of the bone. In contrast to the plant extract-treated groups, which showed a significant increase in strength, when compared to disease approaching that of both the standard and normal control groups. The steroid was the real cause of osteoporosis, as it created an evident imbalance in the rhythm between bone resorption and bone formation, thus causing an increased fracture risk. Steroids adversely affect bones in different ways, where they inhibit the synthesis of collagen and differentiation of osteoblast cells, as well as induce osteoblast cell apoptosis and enhance osteoclast maturation [39]. They mainly act by triggering oxidative stress that, at the cellular level, expresses a wide spectrum of responses, ranging from proliferation to the growth of osteoclast cells, differentiation arrest of osteoblast cells, senescence, cell death by activating major signaling pathway (NF) kB, nitrogen-activated protein kinases, p53, and heat shock factor, with the ultimate occurrence of osteoporosis [40,41]. 

The *Matricaria chamomilla* flower is a rich source of secondary metabolites, particularly flavonoids, sesquiterpenes, coumarins, and glycoside; thus, it effectively prevents oxidative stress-mediated osteoporotic bone damage. The flavonoids previously identified in the plant showed a promising contribution in the alleviation of oxidative stress. Rutin showed promising anti-osteoporotic activity, acting as a drug lead in the alleviation of human osteoporosis, owing to its antioxidant activity. Additionally, it effectively increases the bone mineral density (BMD), in addition to increasing bone trabecula thickness and density, thus keeping it in regular array [42].

Besides, apigenin, kaempferol, and luteolin are reported to prohibit bone loss. Apigenin was believed to be an effective key scavenger invading the free radical produced by the steroid. It is also known to play a major role in differentiation and growth of osteoblast cells. Being a potent antioxidant, apigenin plays a major role in bone formation and resorption process in oxidative stress-induced osteoporosis. This free radical has biochemical link in decreasing mineral concentration and plasma antioxidant levels because the free radical promotes osteoporotic condition by increasing the lipid peroxidation and osteoblastic cell apoptosis; it has also been said that apigenin mediates the cell signaling pathways in bone formation [43]. Additionally, apigenin falls in a phytoestrogen category and seems to act as estrogen on the estrogen receptors on bone and alkaline phosphatase enzymes [44]. Moreover, quercetin showed an in vitro anabolic effect, thus affecting osteoblast proliferation and differentiation, as well as mineralization. It stimulates osteoblast differentiation through the ERK- and ER-mediated pathways. In addition, it activates the anti-inflammatory mechanism, thus resulting in the inhibition of osteoclast differentiation and function. Meanwhile, luteolin previously displayed a pronounced reduction in the differentiation of both bone marrow mononuclear and Raw264.7 cells into osteoclasts, thus inhibiting bone resorption [45].

It is worth highlighting that cathepsin K represents an effective collagenase and predominating papain-like cysteine protease, which is expressed in osteoclasts. Basically, deficiency in cathepsin K in humans and animals reflected a central role of this protease in bone resorption and, thus, have rendered the enzyme as a novel therapeutic target for treating osteoporosis and other disorders that are characterized by an elevation in bone resorption [46]. Thus, molecular docking was performed on the previously identified major phenolic secondary metabolites obtained from *Matricaria chamomilla* L., in order to examine their potential inhibitory effect on cathepsin K. Results showed that all of the examined phenolic compounds showed considerable inhibitory activity on cathepsin K to varying degrees. Rutin showed the best fitting activity within the active site of the tested protein, followed by luteolin-7-*O*-β-glucoside and apigenin-7-*O*-β-glucoside, as evidenced by their free binding energies. From an ADMET/TOPKAT in silico study, it was clear that most of the compounds showed variable pharmacodynamic and pharmacokinetic properties that could be improved, in order to enhance bioavailability. In addition, their toxicity should be carefully monitored by controlling their doses during their incorporation in dosage forms.

## 5. Conclusions

This study shed light on the prophylactic effects of the ethanol extract of the flowers of *Matricaria chamomilla*, regarding its protective effect on bones in steroid-induced osteoporosis. Herein, the corticosteroid induced an osteoporotic condition via increasing oxidative stress, which, in turn, increased the osteoblastic cell number and bone desorption. This is mainly attributed to the richness of the plant with phenolic compounds, particularly flavonoids. This was further ascertained by a molecular modelling study that reflected the pronounced effect of the flavonoids on cathepsin K. However, from an ADMET/TOPKAT in silico study showed variable pharmacodynamic and pharmacokinetic properties exhibited by the examined compounds, which that could be improved to enhance bioavailability during incorporation in different dosage forms. Thus, from the data illustrated in this study, it can be concluded that this plant extract showed potential therapeutic benefits on osteoporosis. However, additional preclinical studies are required to illustrate the mechanism and cell signaling pathways of the produced action of the expected bioactive compounds, as revealed from in silico and in vitro studies, Anyway, micro-computerized tomography (MicroCT) of trabecular and cortical bones and static histomorphometry for osteoblast and osteoclast parameters, as well as the evaluation of serum procollagen 1 intact N-terminal (P1NP), C-terminal telopeptide I, carboxy-terminal collagen I crosslinks (CTX-I), and tartrate-resistant acid phosphatase (TRAP) 5b, are highly recommended to confirm the obtained results.

## Figures and Tables

**Table 1:** Effect of *Matricaria chamomilla* flowers total ethanol extract (MFT) on bone breaking strength (Kg/cm^2^), bone thickness (mm), and bone length (cm).

Group		Bone Breaking Strength (Kg/cm^2^)	Bone Thickness (mm)	Bone Length (cm)
1	Normal control	4.3 ± 0.14 ***	5.51 ± 0.14 ***	3.5 ± 0.08
2	Diseased control (Methyl prednisolone)	3.6 ± 0.23	3.30 ± 0.17	3.5 ± 0.03
3	Standard control (Alendronate)	3.8 ± 0.03 **	4.70 ± 0.12 **	3.5 ± 0.04
4	MFT (200 mg/kg)	3.6 ± 0.05 *	3.9 ± 0.12 *	3.6 ± 0.05 *
5	MFT (400 mg/kg)	4.2 ± 0.27 **	4.8 ± 0.18 **	3.6 ± 0.06 *

Values are expressed as mean ± SEM (*n* = 6). Significantly different from the diseased group at *** *p* < 0.001, ** *p* < 0.01, and * *p* < 0.05.

**Table 2:** Free binding energies (kcal/mol) of the major polyphenolic compounds of *Matricaria chamomilla* flowers in the active site of cathepsin K using in silico studies.

Compound	Cathepsin K	Number of Formed Hydrogen Bonds with the Amino Acid Residues
Apigenin (1)	−32.82	2; Gln19 and Tyr67
Apigenin-7-*O*-β-glucoside (2)	−39.57	2; Gln19 and Trp177
Quercetin (3)	−28.64	1; Cys25
Rutin (4)	−54.19	5; Glu59, Tyr67, Cys25, His159
Luteolin (5)	−28.34	2; Gln19 and Gly66
Luteolin-7-*O*-β-glucoside (6)	−41.91	4; Cys25, Tyr67, His159, Gln19
Naringenin (7)	−33.51	5; Asp61, Gly66, Cys25, Leu157, Tyr67
Umbelliferone (8)	−22.31	3; Gly66, Cys25, Tyr67
Herniarin (9)	−20.70	2; Cys25
Caffeic acid (10)	−23.85	3; Gly66, Cys25, Gly64
Chlorogenic acid (11)	−36.73	3; Gly66 and Asp61
Co-crystalized ligand (NFT)	−67.16	3; Cys25, Gly66, Asp61, Tyr67

NFT: *N*-(2-aminoethyl)-*N*~2~-{(1S)-1-[4′-(aminosulphonyl)biphenyl-4-YL]-2,2,2-triflouroethyl}-*_L_*-Leucinamide.

**Table 3:** Absorption, distribution, metabolism, excretion, and toxicity (ADMET) properties of the major polyphenolic compounds of *Matricaria chamomilla* flowers.

Compounds	Absorption Level	Solubility Level	BBB Level	PPB Level	CPY2D6	Hepatotoxic	Alog p98	PSA-2D
Apigenin (1)	0	3	3	True	NI	True	1.00	81.65
Apigenin-7-*O*-β-glucoside (2)	3	4	4	False	NI	True	−0.93	161.95
Quercetin (3)	1	4	4	True	NI	True	−0.48	119.76
Rutin (4)	3	3	4	False	NI	True	−2.57	263.08
Luteolin (5)	0	3	3	True	NI	True	0.76	102.46
Luteolin-7-*O*-β-glucoside (6)	3	4	4	False	NI	True	−1.71	203.59
Naringenin (7)	0	3	3	False	NI	True	1.67	85.16
Umbelliferone (8)	0	3	3	False	NI	True	0.95	43.53
Herniarin (9)	0	3	2	True	Inhibitor	True	1.88	35.16
Caffeic acid (10)	0	4	3	False	NI	False	−0.03	76.23
Chlorogenic acid (11)	3	4	4	False	NI	False	−1.81	164.91
Co-crystalized ligand (NFT)	1	1	4	True	NI	False	2.96	127.15

The numbers 0, 1, 2, and 3 indicate good, moderate, low, and very low absorption, respectively; 0, 1, 2, 3, 4, and 5 indicate extremely low, very low but possible, low, good, optimal, and too soluble, respectively; 0, 1, 2, 3, and 4 denote very high, high, medium, low, and undefined, penetration via BBB, respectively. PBB, plasma protein binding; FALSE means less than 90%; TRUE means more than 90%; NI: non-inhibitor; False: non-toxic; True: toxic.

**Table 4:** TOPKAT prediction of the major polyphenolic compounds of *Matricaria chamomilla* flowers.

Compounds	Ames Prediction	Rat Oral LD50	Rat Chronic LOAEL	Skin Irritancy	Ocular Irritancy	Rat Female FDA	Rat Male FDA
Apigenin (1)	Non-mutagen	0.164	0.045	None	Moderate	Carcinogen	Non-carcinogen
Apigenin-7-*O*-β-glucoside (2)	Non-mutagen	0.475	0.012	Mild	Moderate	Non-carcinogen	Non-carcinogen
Quercetin (3)	Non-mutagen	0.206	0.142	None	Moderate	Carcinogen	Non-carcinogen
Rutin (4)	Non-mutagen	0.505	0.067	None	Mild	Non-carcinogen	Non-carcinogen
Luteolin (5)	Non-mutagen	0.195	0.073	None	Moderate	Carcinogen	Non-carcinogen
Luteolin-7-*O*-β-glucoside (6)	Non-mutagen	0.518	0.040	None	Moderate	Non-carcinogen	Non-carcinogen
Naringenin (7)	Non-mutagen	1.012	0.076	None	Mild	Carcinogen	Non-carcinogen
Umbelliferone (8)	Non-mutagen	0.610	0.031	Mild	Mild	Carcinogen	Carcinogen
Herniarin (9)	Non-mutagen	0.622	0.018	Mild	Mild	Carcinogen	Carcinogen
Caffeic acid (10)	Non-mutagen	1.125	0.132	None	Mild	Carcinogen	Non-carcinogen
Chlorogenic acid (11)	Non-mutagen	1.363	0.029	Mild	Mild	Non-carcinogen	Carcinogen
Co-crystalized ligand (NFT)	Non-mutagen	7.1897	0.047	None	Mild	Non-carcinogen	Non-carcinogen

Rat oral LD50 and rat chronic LOAEL are measured in g/kg b.wt

## Data Availability

Data are available in the manuscript.

## References

[1] Kasturi G.C., Cifu D.X., Adler R.A. (2009). A review of osteoporosis: Part I. Impact, pathophysiology, diagnosis and unique role of the physiatrist. PM&R.

[2] Wu A.-M., Chi Y.-L., Ni W.-F. (2013). Vertebral compression fracture with intravertebral vacuum cleft sign: Pathogenesis, image, and surgical intervention. Asian Spine J..

[3] Gilman M.T.A. (2011). Goodman Gilman’s The Pharmacological Basis of Therapeutics.

[4] Kanis J.A., Pitt F. (1992). Epidemiology of osteoporosis. Bone.

[5] Fitzpatrick L.A. (2002). Secondary causes of osteoporosis. Mayo Clin. Proc..

[6] Jung W.-W. (2014). Protective effect of apigenin against oxidative stress-induced damage in osteoblastic cells. Int. J. Mol. Med..

[7] Diemar S.S., Sejling A.-S., Eiken P., Andersen N.B., Jørgensen N.R. (2019). An explorative literature review of the multifactorial causes of osteoporosis in epilepsy. Epilepsy Behav..

[8] Mauck K.F., Clarke B.L. (2006). Diagnosis, screening, prevention, and treatment of osteoporosis. Mayo Clin. Proc..

[9] Cosman F., Nieves J.W., Dempster D.W. (2017). Treatment sequence matters: Anabolic and antiresorptive therapy for osteoporosis. J. Bone Min. Res..

[10] Veldurthy V., Wei R., Oz L., Dhawan P., Jeon Y.H., Christakos S. (2016). Vitamin D, calcium homeostasis and aging. Bone Res..

[11] Youssef F.S., Eid S.Y., Alshammari E., Ashour M.L., Wink M., El-Readi M.Z. (2020). *Chrysanthemum indicum* and *Chrysanthemum morifolium*: Chemical composition of their essential oils and their potential use as natural preservatives with antimicrobial and antioxidant activities. Foods.

[12] Orey C. (2019). The Healing Powers of Essential Oils: A Complete Guide to Nature’s Most Magical Medicine.

[13] Ross S.M. (2013). Generalized anxiety disorder (GAD): Efficacy of standardized Matricaria recutita (German chamomile) extract in the treatment of generalized anxiety disorder. Holist. Nurs. Pract..

[14] Murti K., Panchal M.A., Gajera V., Solanki J. (2012). Pharmacological properties of Matricaria recutita: A review. Pharmacologia..

[15] Mann C., Staba E.J. (1986). Herbs, Spices, and Medicinal Plants: Recent Advances in Botany, Horticulture, and Pharmacology.

[16] Kim T.-H., Jung J.W., Ha B.G., Hong J.M., Park E.K., Kim H.-J., Kim S.-Y. (2011). The effects of luteolin on osteoclast differentiation, function in vitro and ovariectomy-induced bone loss. J. Nutr. Biochem..

[17] Shi X., Li C., Wan Q., Li A., Wang H., Liu K. (2014). Drynaria total flavonoids decrease cathepsin K expression in ovariectomized rats. Genet. Mol. Res..

[18] Karbalaei D., Nourafshan A. (2009). Antiulcerogenic effects of *Matricaria chamomilla* extract in experimental gastric ulcer in mice. Iran. J. Med. Sci..

[19] Chitme H., Muchandi I., Burli S. (2009). Effect of Asparagus racemosus Willd root extract on ovariectomized rats. Open Nat. Prod. J..

[20] Salman S., Kumbasar S., Hacimuftuoglu A., Ozturk B., Seven B., Polat B., Gundogdu C., Demirci E., Yildirim K., Akcay F. (2012). The effect of metyrosine/prednisolone combination to oophorectomy-induced osteoporosis. Iran. J. Reprod. Med..

[21] Song L. (2017). Calcium and bone metabolism indices. Adv. Clin. Chem..

[22] Li C.S., Deschenes D., Desmarais S., Falgueyret J.-P., Gauthier J.Y., Kimmel D.B., Léger S., Massé F., McGrath M.E., McKay D.J. (2006). Identification of a potent and selective non-basic cathepsin K inhibitor. Bioorg. Med. Chem. Lett..

[23] Janibekov A.A., Youssef F.S., Ashour M.L., Mamadalieva N.Z. (2018). New flavonoid glycosides from two *Astragalus* species (Fabaceae) and validation of their antihyperglycaemic activity using molecular modelling and in vitro studies. Ind. Crops Prod..

[24] Altyar A.E., Ashour M.L., Youssef F.S. (2020). Premna odorata: Seasonal metabolic variation in the essential oil composition of its leaf and verification of its anti-ageing potential via in vitro assays and molecular modelling. Biomolecules.

[25] Youssef F.S., Ovidi E., Musayeib N.M.A., Ashour M.L. (2021). Morphology, anatomy and secondary metabolites investigations of Premna odorata Blanco and evaluation of its anti-tuberculosis activity using in vitro and in silico studies. Plants.

[26] Gupta V., Mittal P., Bansal P., Khokra S.L., Kaushik D. (2010). Pharmacological potential of Matricaria recutita—A review. Int. J. Pharm. Sci. Drug Res..

[27] Exner J., Reichling J., Becker H. (1980). Flavonoids in *Matricaria chamomile*. Planta Medica.

[28] Kunde R., Isaac O. (1979). Über die Flavone der Kamille (*Matricaria chamomilla* L.) und ein neues acetyliertes Apigenin–7–glucoside. Planta Med..

[29] Tyihak E., Sarkany-Kiss J., Verzar-Petri G. (1962). Phytochemical investigation of apigenin glycosides of *Matricaria chamomilla*. Pharmazie.

[30] Singh O., Khanam Z., Misra N., Srivastava M.K. (2011). Chamomile (*Matricaria chamomilla* L.). An overview. Pharmacog. Rev..

[31] Xie X.-Y., Chen F.-F., Shi Y.-P. (2014). Simultaneous determination of eight flavonoids in the flowers of *Matricaria chamomilla* by high performance liquid chromatography. J. AOAC Int..

[32] Sambrook P. (2005). How to prevent steroid induced osteoporosis. Ann. Rheum. Dis..

[33] Weiss M.J., Henthorn P.S., Lafferty M.A., Slaughter C., Raducha M., Harris H. (1986). Isolation and characterization of a cDNA encoding a human liver/bone/kidney-type alkaline phosphatase. Proc. Natl. Acad. Sci. USA.

[34] Sharma U., Pal D., Prasad R. (2014). Alkaline phosphatase: An overview. Ind. J. Clin. Biochem..

[35] Lowe D., Sanvictores T., John S. (2017). Alkaline phosphatase. Treasure Island.

[36] Bonjour J.-P. (2011). Calcium and phosphate: A duet of ions playing for bone health. J. Am. Coll. Nutri..

[37] Castiglioni S., Cazzaniga A., Albisetti W., Maier J.A. (2013). Magnesium and osteoporosis: Current state of knowledge and future research directions. Nutrients.

[38] Huh J.H., Choi S.I., Lim J.S., Chung C.H., Shin J.Y., Lee M.Y. (2015). Lower serum creatinine is associated with low bone mineral density in subjects without overt nephropathy. PLoS ONE.

[39] Ilias I., Zoumakis E., Ghayee H., Feingold K.R., Anawalt B., Boyce A. (2018). An overview of glucocorticoid induced osteoporosis. Endocrinology Book.

[40] Domazetovic V., Marcucci G., Iantomasi T., Brandi M.L., Vincenzini M.T. (2017). Oxidative stress in bone remodeling: Role of antioxidants. Clin. Cases Min. Bone Metabol..

[41] Abdollahi M., Larijani B., Rahimi R., Salari P. (2005). Role of oxidative stress in osteoporosis. Clin. Pract..

[42] Wang Q., Huo X., Wang J., Wang D., Zhu Q., Liu B., Xu L. (2017). Rutin prevents the ovariectomy-induced osteoporosis in rats. Eur. Rev. Med. Pharmacol. Sci..

[43] Zhou X., Wang F., Zhou R., Song X., Xie M. (2017). Apigenin: A current review on its beneficial biological activities. J. Food Biochem..

[44] Yao L., Fan Z., Han S., Sun N., Che H. (2020). Apigenin attenuates the allergic reactions by competitively binding to ER with estradiol. Front. Pharmacol..

[45] Badole S., Kotwal S. (2014). Equisetum arvense: Ethanopharmacological and Phytochemical review with reference to osteoporosis. Int. J. Pharm. Sci. Health Care.

[46] Brömme D., Lecaille F. (2009). Cathepsin K inhibitors for osteoporosis and potential off-target effects. Expert Opin. Investig. Drugs.

